# Regulation of *Drosophila* Long-Term Courtship Memory by Ecdysis Triggering Hormone

**DOI:** 10.3389/fnins.2021.670322

**Published:** 2021-04-22

**Authors:** Sang Soo Lee, Michael E. Adams

**Affiliations:** ^1^Neuroscience Graduate Program, University of California, Riverside, Riverside, CA, United States; ^2^Department of Molecular, Cell, and Systems Biology, University of California, Riverside, Riverside, CA, United States; ^3^Department of Entomology, University of California, Riverside, Riverside, CA, United States

**Keywords:** long-term memory, *Drosophila* courtship conditioning, ecdysis triggering hormone, juvenile hormone, mushroom body, hormonal convergence

## Abstract

Endocrine state is an important determinant of learning and memory in animals. In *Drosophila*, rejection of male courtship overtures by mated females leads to an aversive response manifested as courtship memory. Here we report that ecdysis triggering hormone (ETH) is an obligatory enabler of long-term courtship memory (LTM). ETH deficiency suppresses LTM, whereas augmented ETH release reduces the minimum training period required for LTM induction. ETH receptor knockdown either in the mushroom body (MB) γ lobe or in octopaminergic dorsal-anterior-lateral (DAL) neurons impairs memory performance, indicating its direct action in these brain areas. Consistent with these findings, brain exposure to ETH mobilizes calcium in MB γ lobe neuropils and DAL neurons. ETH receptor (ETHR) knockdown in the corpus allatum (CA) to create juvenile hormone (JH) deficiency also suppresses LTM, as does knockdown of the JH receptor Met in the MB γ lobe, indicating a convergence of ETH and JH signaling in this region of the brain. Our findings identify endocrine-enabled neural circuit components in the brain that are critical for persistent behavioral changes resulting from aversive social experience.

## Introduction

Social experiences profoundly influence internal states and subsequent behavioral patterns in a broad range of animal species, including the fruit fly, *Drosophila melanogaster* (Chabaud et al., 2009; Shohat-Ophir et al., 2012). Such experience-based modulation of social interactions in fly communities may be critical for survival and propagation (Siegel and Hall, 1979; Svetec and Ferveur, 2005; Krupp et al., 2008). An example of social context-dependent behavioral plasticity in *Drosophila* is courtship memory, whereby rejection of male courtship overtures by mated females suppresses subsequent courtship attempts, even toward receptive virgin female partners (Siegel and Hall, 1979; McBride et al., 1999). Previous studies have reported structural and molecular components of courtship memory that are shared with olfaction-based memories. In particular, both are triggered by chemosensory inputs, chief among which are pheromones. However, courtship memory cannot be explained by olfactory influences alone, since males exposed to mated females experience multimodal aversive sensory inputs, in particular behavioral cues associated with rejection by the female during training (Keleman et al., 2012; Lee et al., 2017). Furthermore, distinct neural pathways involved in memory recall depend on the reproductive state (mated vs. virgin) of target females during the post-training test session (Ejima et al., 2007; Keleman et al., 2012). This type of associative memory may be influenced by additional internal components, among which could be endocrine state. While the steroid ecdysone promotes both short- and long-term courtship memories through distinct neural pathways (Ishimoto et al., 2009, 2013), how hormonal influences regulate learning and memory processes under courtship conditioning is still poorly understood.

Hormonal cascades drive ecdysis, a critically important innate behavior required for shedding of old cuticle and advancement to the next developmental stage (Zitnan et al., 2003). This fixed action pattern is initiated by circulating peptides called ecdysis triggering hormones (ETHs). ETHs, released by epitracheal gland Inka cells, orchestrate downstream peptidergic neuronal ensembles leading to sequential behaviors (Zitnan et al., 1996; Kim et al., 2006, 2015). ETH stimulates target cells *via* ETH receptor (ETHR)-mediated mobilization of calcium (Kim et al., 2006; Lee et al., 2017). Interestingly, Inka cells and associated transcripts of ETH and ETHRs persist into the adult stage of *Drosophila* (Park et al., 2002; Graveley et al., 2011). The adult roles of ETH signaling are just beginning to be unraveled and include reproductive fitness along with learning and memory.

We recently reported that ETH has an allatotropic function; i.e., promotion of juvenile hormone biosynthesis in adult *Drosophila* (Lee et al., 2017; Meiselman et al., 2017). ETHRs expressed in the corpus allatum (CA), the sole source of juvenile hormone (JH), regulate intracellular calcium levels in response to ETH. In females, ETH-driven JH synthesis is essential for reproductive fitness, while this hormonal cascade also affects male reproduction, possibly through regulation of accessory gland protein synthesis.

Another important functional role for the adult ETH-JH signaling cascade is regulation of short-term memory (STM) retention of males following courtship conditioning (Lee et al., 2017), whereby JH targets dopaminergic (DA) neurons. In this study, we provide evidence that hormonal states established by ETH and JH are also essential for long-term memory (LTM) performance following extended courtship conditioning. In this context, we report here that ETH actions on brain circuits are both indirect and direct: (1) ETH acts directly on dorsal anterior lateral (DAL) neurons and in concert with JH on mushroom body (MB) neurons, and (2) ETH acts indirectly through promotion of JH production. Our results provide evidence for a critical role of endocrine state in enabling social context-dependent behavioral modification and provide an approach for identification of neural circuit components through selective hormone receptor silencing.

## Materials and Methods

### Fly Strains

*Drosophila melanogaster* stocks were maintained at 25°C on standard cornmeal-agar media under a 12-h light/dark (LD) regimen, except for certain experimental manipulations. *Canton-S* flies were used as wild-type. To reduce genetic background, all lines in this study were outcrossed to a wild-type line, *wCS10* for at least five generations. *JHAMT-GAL4* was obtained from B. Dauwalder (University of Houston) (Wijesekera et al., 2016). *ETH-GAL4* was obtained from D. Anderson (California Institute of Technology). *ETH-GS-GAL4*, designated here as “*EUG8*,” was described previously (Cho et al., 2014). *ETHR-GAL4* lines were obtained from B. White (National Institute of Mental Health) (Diao et al., 2016). *UAS-hid,rpr* was provided by P. Taghert (Washington University). An *elav-GS-GAL4* driver *GSG301* was provided by R. Davis (The Scripps Research Institute, Florida) (Osterwalder et al., 2001). *4.59* line was provided from U. Heberlein (Janelia Research Campus) (Kaun et al., 2011). *UAS-ETHR RNAi* lines (*ETHRi-Sym*, *ETHRi-IR2*) targeting independent sequences of the receptor are described previously (Kim et al., 2015). *UAS-Met RNAi* and *UAS-gce RNAi* lines were obtained from the Vienna *Drosophila* Research Center. The following Split-GAL4 lines were obtained from the Janelia Research Campus: *MB009B*, *MB011B*, *MB093C*, *MB131B*, and *MB399B*. Specificities of these driver lines are described on the Janelia Research “FlyLight Split Gal4 Driver Collection” website^1^. The following lines were obtained from the Bloomington Stock Center: *OK107*, *201Y*, *c739*, *1471*, *TH-GAL4*, *Orco-GAL4*, *Dilp2-GAL4*, *G0338*, *G0431*, *UAS-dTrpA1*, *UAS-mCD8-GFP*, *UAS-Shi^*ts*^*, and *UAS-GCaMP5*.

### Courtship Conditioning and Statistical Analysis

Animals were prepared for courtship conditioning as previously described (Lee et al., 2017). In short, males of appropriate strain were collected during the pupal stage and individually housed in clean glass tubes containing fresh food for 4 days posteclosion to prevent pre-test social interaction. To prevent anesthetic effects, we used a mouth aspirator to transfer or collect animals. Trainer females were prepared by housing day 3–5 virgin Canton-S females with *Canton-S* males in food-containing glass tubes overnight. Immobilized tester females were prepared by decapitating day 4–5 virgin *Canton-S* females with fine scissors under CO_2_ anesthesia. Protocols for long-term courtship conditioning followed those described previously (Keleman et al., 2007; Ishimoto et al., 2009), with some modifications. Briefly, a 4-day old male was paired with a mated *Canton-S* female for 5 h in the food chamber (2.0 ml tube). Trained (or sham-trained) males were individually housed in a fresh food tube for 24 h prior to testing, whereupon courtship activity of a trained (or sham-trained) male toward an immobilized (decapitated) tester female was recorded for 10 min using a high-frame digital camcorder (SONY HDR-XR260V). All assays were manually scored for courtship index, blind to the genotype and to the extent possible, the experimental condition. Positive contributions to courtship index (CI), defined as the percentage of time a male performs courtship behavior over a 10 min interval, included all courtship activities such as orienting, tapping, singing, licking, and attempting to copulate.

Courtship memory is quantified through computation of a memory performance index (MPI), which is the **relative** reduction of CI of individual trained male (^*i*^CI_*T*_) from the mean CI of sham-trained males (^*m*^CI_*S*_):

MPI=CISm-CITiCISm

Statistical analyses were carried out using the Mann–Whitney *U* test for comparison between CI of trained and sham-trained males. For comparison between individual MPI of each control group and test group, a custom R script was developed to perform a 100,000 random permutation test (Keleman et al., 2007; Lee et al., 2017). Asterisks (^∗^) indicate statistical difference between MPI of test males and that of each control group, except in cases indicated in figure legends (*^∗^p* < 0.05; *^∗∗^p* < 0.01; *^∗∗∗^p* < 0.001; ns, not significant).

### Thermogenetic Controls

For TARGET experiments shown in Figure 2C, flies were raised at 19°C during the juvenile period, then transferred within a day of eclosion to 31°C for 4 days until training. Immediately following training at room temperature (20–21°C) for 5 h, flies were again transferred to 31°C for 24 h until testing at room temperature. For experiments depicted in Figure 4B, flies were raised at 19°C during the juvenile period and individually housed at 19°C for 6 days following eclosion. Flies were then transferred to 31°C until initiation of courtship conditioning, which was conducted at room temperature (20–21°C).

**FIGURE 1:**
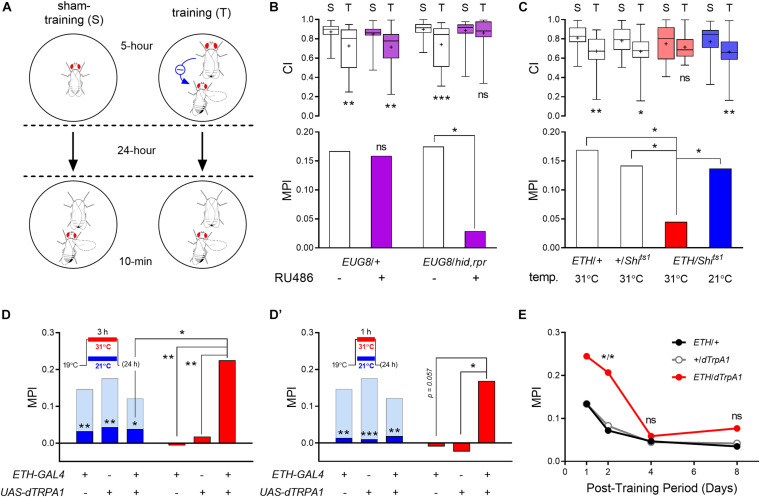
ETH signaling regulates *Drosophila* courtship LTM. **(A)** Schematic of the long-term courtship conditioning procedure. **(B)** ETH deficiency was created by ablation of Inka cells through conditional ectopic expression of apoptosis genes *hid* and *rpr* under the control of RU486-activated GeneSwitch GAL4. Transgenic animals show significant reduction of MPI when treated with RU486 as compared to controls (*n* = 54–56). **(C)** Conditional block of vesicular secretion by Inka cells was achieved through expression of temperature-dependent dynamin mutant *shibire* in Inka cells. Courtship conditioning was conducted at the restrictive temperature, while testing occurred at room temperature. Transgenic flies exhibit less suppression of courtship activities following training than those of control groups (*n* = 48–52). Activation of adult Inka cells during 3- **(D)**, and 1-h **(D′)** training with a mated female at 31°C utilizing dTrpA1 overexpression. *ETH-GAL4*/*UAS-dTrpA1* males show 22.5% **(D)** and 16.9% **(D′)** courtship suppression following 3- and 1-h training at 31°C, respectively, compared to sham-trained males (*n* = 60–80). Light blue bars indicate MPI levels attained following a 5-h training protocol at 21°C, whereas dark blue bars depict significant reduction of MPI levels following 3- **(D)** or 1-h **(D′)** training protocols at 21°C. **(E)** Effect of Inka cell activation during 5-h training on persistence of memory performance. Training and sham-training of all animals were performed at 31°C. Asterisks represent statistical differences between data from test groups and GAL4 and UAS genetic control groups (*n* = 64–78). Mann–Whitney *U* test for CI and random permutation test for MPI; *p < 0.05, **p < 0.01, and ***p < 0.001. ns, no significance.

For experiments involving *shibire* (Figure 1C), flies were raised at 19°C during the juvenile period and housed at 31°C for 4 days following eclosion. For the training session and 24-h post-training interval, flies were maintained either at 21 or 31°C; testing was performed at room temperature.

**FIGURE 2:**
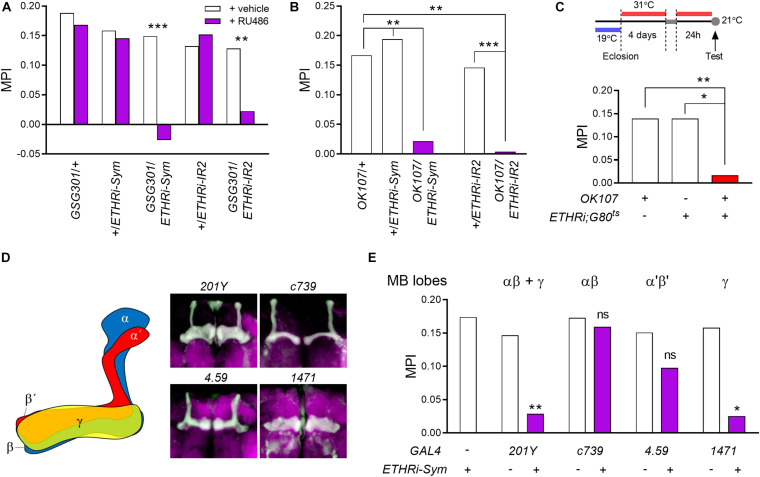
ETHR expression in the MB γ lobe is essential for courtship LTM performance. **(A)** Conditional knockdown of ETHR-encoding genes was achieved using the drug-inducible pan-neuronal GeneSwitch system (*GSG301*) by feeding RU486 for 3 days to day 4 males. LTM is lost in both *GSG301*/*UAS-ETHR RNAi-Sym* and *GSG301*/*UAS-ETHR RNAi-IR2* males (*n* = 60–64). **(B)** ETHR knockdown using two independent RNAi lines in MB neurons sharply reduces MPI (*n* = 60–66). **(C)** Conditional ETHR silencing in adult MB neurons results in courtship LTM impairment (*n* = 64–72). **(D,E)** ETHR knockdown in MB γ-containing neurons impairs courtship LTM: **(D)** GAL4 lines utilized in this study: 201Y (αβ and γ), c739 (αβ), 4.59 (α′β′), and 1,471 (γ). Expression patterns were confirmed by crossing *UAS-mCD8-GFP*; **(E)** memory performances of MB lobe-specific ETHR-silenced males (*n* = 64–70). Random permutation test, *p < 0.05; **p < 0.01; and ***p < 0.001; ns, no significance.

For dTrpA1 experiments (Figures 1D,E), male flies were raised at 19°C during the first 4 days of adulthood, then housed at either 31 or 21°C with a mated female for the indicated training periods (3- and 1-h training). After a 24-h interval period at 19°C, memory performance was tested by pairing an individual male with an immobilized (decapitated) virgin female at room temperature.

### Drug Treatments

Drug treatment associated with GeneSwitch experiments was described previously (McGuire et al.,2004a,b). Briefly, flies carrying the transgene *EUG8-GAL4* were fed 200 μM mifepristone (RU486, Sigma) in normal fly food beginning immediately after eclosion for 4 days (Figure 1B), and from day 4 to 7 (Figures 2A, 5A) until courtship conditioning. Ethanol (1.6%) was used as vehicle. The JH analog methoprene (JHA; 1x, 64.4 pmol or 10×, 644 pmol) was applied topically in acetone to the ventral abdomen of day 0 posteclosion males held under cold anesthesia using a Nanoject II (Drummond).

### Immunohistochemistry

Brains dissected from adults 4–5 days after eclosion were fixed in 4% paraformaldehyde at 4°C, washed sequentially with PBS and PBST (0.5% Triton X-100 in PBS), then blocked with 3% normal goat serum (NGS) for 1 h at room temperature. Primary antibody incubation in PBST with 3% NGS at 4°C overnight was followed by incubation in fluorophore-conjugated secondary antibodies overnight at 4°C. Brains were mounted in Aqua Poly/Mount on slides for imaging. Primary antibodies used were rabbit anti-GFP IgG (1:500, Invitrogen) and mouse anti-Bruchpilot (nc82) (1:50, DSHB). Fluorophore-conjugated secondary antibodies were Alexa Fluor 488 goat anti-rabbit (1:200, Invitrogen) and Alexa Fluor 568 goat anti-mouse (1:200, Invitrogen). Confocal images were acquired with a Zeiss LSM 510 microscope. To obtain clearer expression patterns for some GAL4 lines, image colors were inverted using raster graphic software (Adobe Photoshop CC).

### Functional *in vitro* Ca^2+^ Imaging

Ca^2+^ dynamics were monitored *via* functional imaging utilizing the genetically encoded Ca^2+^ reporter GCaMP5 (Akerboom et al., 2012) expressed in specific neuronal populations using the GAL4/UAS system. Brains were extirpated and maintained in ice-chilled fly saline during recordings. Fluorescence responses were visualized with a TILL-Imago CCD camera mounted on an Olympus BX51W1 microscope; Live Acquisition software was used for control of a Polychrome V monochronometer (excitation wavelength 488 nm). Excitation pulses (488 nm, 50 ms duration) were applied at 1 Hz and emission was recorded continuously *via* a 515 long pass filter. Following 2-min of pre-application sampling, *Drosophila* ETH1 (DmETH1) was applied.

## Results

### ETH Signaling Is Required for LTM in Courtship Conditioning

Mature virgin *Drosophila* females are generally receptive to male courtship advances, whereas recently mated females reject mating attempts. Following rejection or exposure to male-specific anti-aphrodisiac pheromones from mated females, males suppress subsequent courtship attempts regardless of female receptivity (Ejima et al., 2007). Furthermore, male courtship suppression toward a new virgin partner can be short- (∼1–2 h following 1-h training) or long-lasting (>24 h after 5–7-h training), depending on the duration of sexual rejection experienced during encounters with unreceptive females (McBride et al., 1999). Altered male behaviors following courtship conditioning for short or longer periods constitute courtship STM and LTM, respectively.

In a previous report, we showed that genetic disruption of the ETH-JH hormonal cascade impairs courtship STM, whereby normal males suppress courtship overtures by ∼30% toward an immobilized virgin female for up to 2 h following a 1-h training interval with a mated female (Lee et al., 2017). To investigate whether disruption of ETH signaling also affects courtship LTM, we applied an extended courtship conditioning protocol (see section “Materials and Methods” for details) (Figure 1A). Following a 5 h training period, we observed suppression of male courtship behavior between 14 and 20%, expressed as MPI when assayed 24 h after completion of training.

We first tested whether ETH deficiency affects LTM by conditionally ablating Inka cells posteclosion in order to avoid lethal ecdysis deficiencies during development (Park et al., 2002). This was accomplished by preparing transgenic flies expressing apoptosis genes (*hid* and *rpr*) specifically in Inka cells using the drug-inducible GeneSwitch driver (*ETH-GS-GAL4*, designated as *EUG8*) (Cho et al., 2014). We found that adult-specific ablation of Inka cells by feeding males (*EUG8 > UAS-hid,rpr*) the GAL4 activator RU486 impairs memory performance. RU486 feeding had no effect on memory performance of a genetic control group (Figure 1B). Likewise, Inka cell-ablated males showed no significant changes in basal courtship activities toward immobilized virgin females; i.e., the CI of sham-trained males was unchanged.

We next used an alternative strategy for creating ETH deficiency by blocking vesicular secretion by Inka cells *via* activation the dominant-negative temperature sensitive dynamin mutant *shibire* (*UAS-Shi^*ts1*^*) (Kitamoto, 2001). Blocking Inka cell release at the restrictive temperature during both maturation and courtship conditioning periods impaired LTM performance without affecting basal courtship activity (Figure 1C). These data indicate that Inka cell secretory activity is essential for normal courtship LTM performance of males.

### Augmented Inka Cell Function Improves Memory Performance

We have shown that ETH deficiency during adulthood suppresses courtship LTM performance (Figure 1). To test whether augmented ETH release from Inka cells during the training period affects memory performance, we expressed the temperature-sensitive cation channel, *Drosophila* TrpA1 (*dTrpA1*) in Inka cells using the *ETH-GAL4* driver. In our extended courtship conditioning protocol (described in the “Materials and Methods” section), male flies require a minimum of 5-h training with a mated female to show significant courtship suppression after 24 h (Figures 1D,D′; light blue bars). Reduction of the training period to 3 h or 1 h caused reduction of MPI to levels below 4% (Figures 1D,D′; dark blue bars).

We asked whether augmented Inka cell activity through dTrpA1 activation could enhance memory performance during shorter training periods. When paired with a mated female for a 3-h training period at the TrpA1 permissive temperature, substantial enhancement of MPI was observed (Figure 1D), reaching *p* < 0.01 compared to GAL4 and UAS controls. When the training period was reduced further to only 1-h, enhancement reached *p* < 0.05 for the UAS control and *p* = 0.057 for the GAL4 control, suggesting that significant enhancement also occurred following this minimal training period. (Figure 1D′). Genetic control groups did not show detectable courtship suppression by 3- or 1-h training at both temperature conditions. No significant difference in basal courtship activity was observed in sham-trained *ETH-GAL4*/*UAS-dTrpA1* males toward an immobilized tester virgin female following heat-activation compared to the genetic control groups (Supplementary Figure 1).

We next asked whether augmented Inka cell activity affects maintenance of memory following long-term courtship conditioning. Although *Drosophila* courtship LTM following 5-h training with a mated female lasts at least several days, the intensity of memory performance decays gradually over this period (McBride et al., 1999). Significant enhancement of MPI for *ETH-GAL4*/*UAS-dTrpA1* males trained for 5 h at 31°C over controls was not observed after 24 h, given the relatively high MPI of controls (see Figure 1E). However, the test male group showed a significant increase of MPI 48-h after training compared to both genetic control groups (Figure 1E). Thus, we conclude that augmented Inka cell activity during the 5-h training extends a high rate of memory performance (>20%) to 48 h after training.

### ETHRs in MB γ Lobe Neurons Regulate Courtship LTM

Although functions of ETH-ETHR signaling in central neurons of juvenile animals have been described (Kim et al., 2006, 2015; Diao et al., 2016; Mena et al., 2016), presence of ETHRs in the adult brain (Supplementary Figure 2) suggests their possible roles in behavioral regulation in adults. In particular, a recent study described expression of ETHRs in several neuronal subtypes of memory-related circuits in the adult brain through cell-specific transcriptome analyses (Crocker et al., 2016). Although their findings suggest low levels of ETHR expression in MB neurons, we used the genotype *ETHR-GAL4 (MH1506)* > *UAS-mCD8-GFP* to identify a subset of cells (approximately 2 μm diameter) in the MB calyx area (Supplementary Figure 2C).

We first asked whether RNA knockdown of *ETHR* in the nervous system affects courtship LTM. We employed adult-specific RNA knockdown of *ETHR* genes using the conditional pan-neuronal GeneSwitch driver *elav-GS-GAL4* (*GSG301*) enabled by RU486 (Osterwalder et al., 2001; Ford et al., 2007). Employing ETHR RNA knockdown with two independent RNAi constructs (Kim et al., 2015) targeting distinct regions common to the *ETHR-A* and *ETHR-B* sequences, we obtained strong suppression of courtship LTM performance (Figure 2A).

To examine the role of ETHR expression in MB neurons, we first used the pan-MB GAL4 driver *OK107* to effect RNA knockdown of ETHRs and observed strong suppression of courtship LTM performance using two dsRNA constructs targeting distinct regions of the ETHR gene (*UAS-ETHR RNAi-Sym* and *UAS-ETHR RNAi-IR2*) (Figure 2B). In contrast, ETHR silencing in MB neurons did not show deficit in STM performance (Supplementary Figure 3A) following 1-h training, suggesting that these two forms of courtship memory arise through independent mechanisms. To confirm the requirement of ETHR expression in posteclosion adult MB neurons for courtship LTM, we applied the temporal and regional gene expression targeting (TARGET) system (McGuire et al.,2004a,b), again using the *OK107* driver and found that RNA knockdown of *ETHR* genes in the entire MB during adulthood also severely impairs courtship LTM (Figure 2C). ETHR silencing did not affect the size or gross morphology of MB lobes when we performed the immunostaining against FasII (Supplementary Figure 3B).

The MB consists of three major classes of neuropils: the αβ, α′β′, and γ (Figure 2D). To more precisely identify specific functional targets of ETH regulating courtship LTM, we performed knockdown of *ETHR* genes using a set of GAL4 drivers specific for the following neuropil areas: αβ and γ (*201Y*), αβ (*c739*), α′β′ (*4.59*), and γ (*1471*). ETHR knockdown using the *201Y* and *1,471* drivers significantly impaired courtship LTM, whereas memory performance was unaffected by ETHR silencing in the αβ lobe (*c739*) (Figure 2E). Although slight suppression of LTM performance occurred following ETHR knockdown with the *4.59* driver, reduction did not reach statistical significance. Taken together, our data thus suggest that ETHR expression in the MB γ lobe is required for courtship LTM performance.

### ETH Signaling Targets Multiple Memory-Related Neurons

Ecdysis triggering hormone receptor expression in the adult brain (Supplementary Figure 2; Crocker et al., 2016) suggests that ETH could target multiple neuronal circuits. We therefore selected an array of Split-GAL4 lines with high regional specificity to test whether ETHR silencing affects courtship LTM: *MB009B* and *MB131B* driving expression in MB γ neurons exclusively (Masek et al., 2015), along with *MB093C*, *MB011B*, and *MB399B* expressing in MB output neurons (MBONs) (Aso et al., 2014). To target the DAL neurons, the drivers *G0338* and *G0431* were employed (Chen et al., 2012).

**FIGURE 3:**
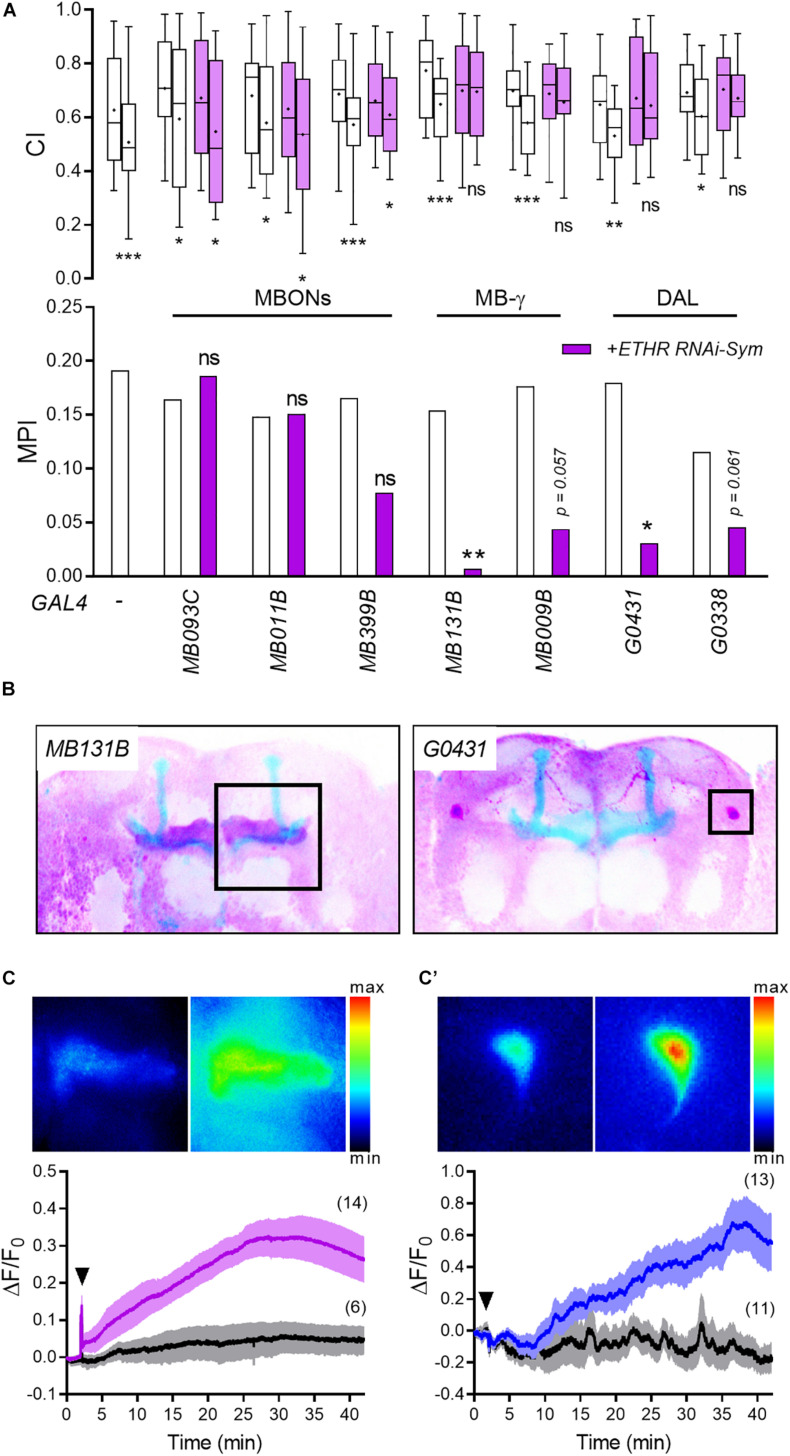
ETH targets specific memory circuit neurons to regulate courtship LTM. **(A)** ETHR knockdown in utilizing an array of candidate neuronal driver lines. Mann–Whitney *U* test for CI and random permutation test for MPI: *p < 0.05; **p < 0.01; ***p < 0.001; ns, no significance. **(B)** Expression patterns of *MB131B* and *G0431* drivers, which show memory defects following ETHR knockdown in panel **(A)**, were confirmed by crossing *UAS-mCD8-GFP*. To improve contrast, colors were inverted: purple, GFP; blue, Fas-II (MB). **(C,C′)** ETH mobilizes Ca^2+^ in the MB γ lobe [**(C)**, *MB131B*/*UAS-GCaMP5*] and DAL neurons [**(C′)**, *G0431*/*UAS-GCaMP5*]. Upper representative images show before (left) and after (right) exposure to 1 μM DmETH1. Bottom: averaged traces of ΔF/F_0_ following saline (black) and 1 μM DmETH1 (purple or blue). Arrowheads represent saline or ETH application. Darker lines show means, and the light envelopes represent SEM. Parentheses indicate the number of recordings.

Consistent with results shown in Figure 2E, RNA knockdown of ETHRs in the MB γ lobe abolished differences in CI between sham-trained and courtship-conditioned males (Figure 3A). MPI reduction following ETHR knockdown *via* the MB γ lobe-specific driver *MB131B*/*UAS-ETHR RNAi-Sym* was significant (*p* < 0.01), whereas the *MB009B*/*UAS-ETHR RNAi-Sym* trended close to significance at the 95% level (*p* = 0.057).

**FIGURE 4:**
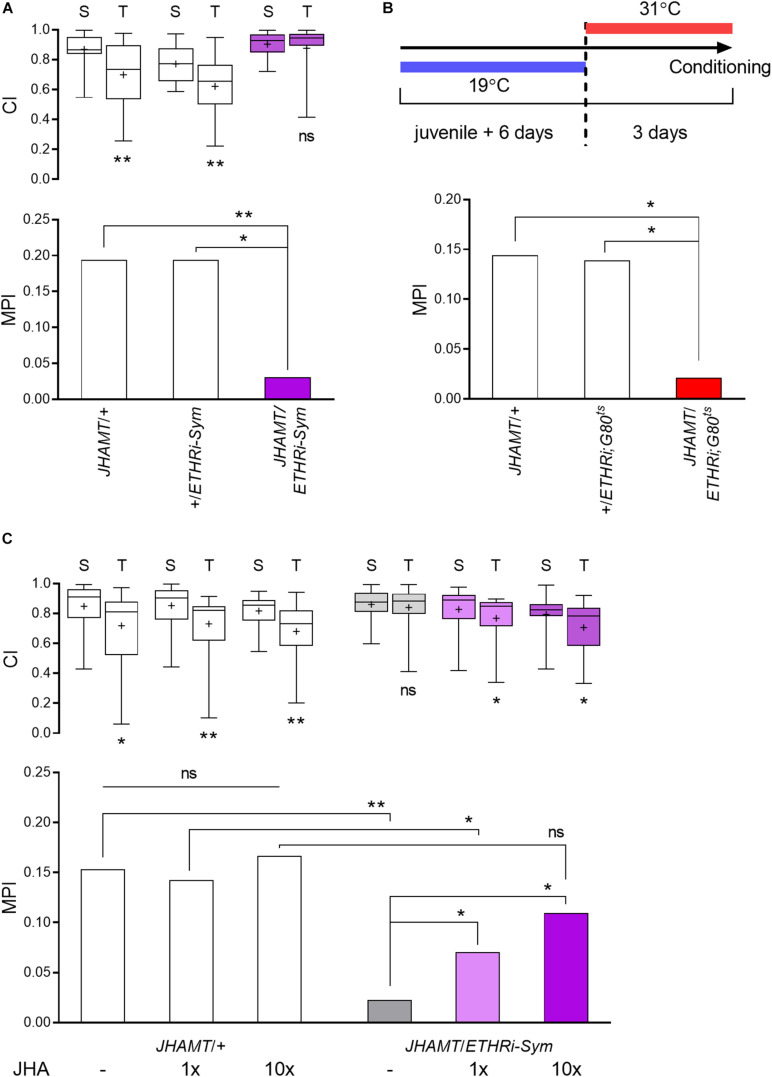
ETH-JH hormonal cascade is essential for courtship LTM. **(A)** The role of ETH-JH signaling in courtship LTM was examined following ETHR knockdown in the CA to create JH deficiency. CA-specific ETHR silencing leads to memory defect, whereas both genetic control groups are normal (*n* = 50–64). **(B)** Conditional ETHR silencing in the CA *via* the TARGET system. Adult-specific ETHR silencing causes a significantly less memory performance by training compared to genetic controls (*n* = 58–66). **(C)** Rescue of memory phenotype in ETHR-silenced males by topical treatment with the JH analog methoprene (*n* = 48–62): “–,” vehicle (acetone); “+,” methoprene (1x, 64.4 pmol; 10x, 644 pmol per animal). Vehicle-treated *JHAMT-GAL4*/*UAS-ETHR RNAi* males suppress courtship by 2.3%, while and 1x JHA-treated males show 7.0% suppression of courtship; the MPI of 10x JHA-treated males is 10.9%. Mann–Whitney *U* test for CI and random permutation test for MPI: *p < 0.05; **p < 0.01; ns, no significance.

**FIGURE 5:**
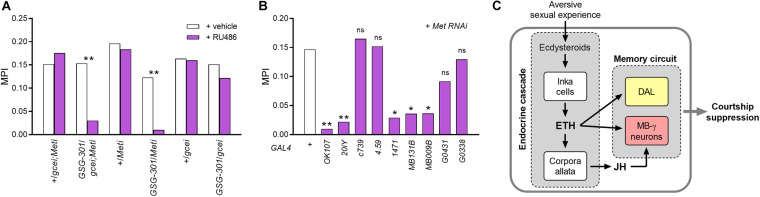
Hormonal convergence for courtship LTM regulation. **(A)** Loss of LTM in *GSG301*/*UAS-gce RNAi;UAS-Met RNAi* and *GSG301*/*UAS-Met RNAi* males, whereas *GSG301*/*UAS-gce RNAi* males does not show significantly different memory performance compared to vehicle-treated animals (*n* = 62–68). **(B)** RNA knockdown of Met in ETH-targeted neurons: MB γ lobe-containing neuronal drivers (*OK107*, *201Y*, *1,471*, *MB131B*, and *MB009B*) significantly impair LTM performance by Met-silencing (*n* = 60–62). Random permutation test, *p < 0.05; **p < 0.01; ns, no significance. **(C)** A model for the endocrine system function in courtship memory induced by long-term conditioning. Proposed model as described in the text depicting the direct and indirect pathways of ETH signaling and hormonal convergence of ETH and JH in regulation of *Drosophila* courtship LTM in response to repeated failure in courtship.

We also observed that ETHR knockdown using both DAL neurons drivers (*G0431* and *G0338*) abolished CI differences between sham-trained and courtship-conditioned males (Figure 3A). While memory performance (MPI) using the DAL neuronal driver *G0431* was impaired significantly (*p* < 0.05), results using the *G0338* driver trended toward significance (*p* = 0.061).

While the CI comparisons were made with identical genotypes (sham-trained GAL4 > UAS vs trained GAL 4 > UAS), the MPI comparisons were made between different genotypes (GAL4 control vs Gal4 > UAS). This appears to have introduced an additional variability factor that may account for loss of significance at the *p* < 0.05 threshold for the MB009B-Gal4 and G0338-Gal4 drivers.

In contrast, ETHR knockdown using all MBON driver lines did not abolish CI differences between sham-trained and courtship-conditioned males and led to no statistically significant differences in MPI compared to genetic control groups. Adult-specific RNA knockdown of ETHRs in olfactory (*Orco-GAL4*), dopaminergic (*TH-GAL4*), and insulin-producing cells (*Dilp2-GAL4*) also did not result in significant changes in courtship LTM performance (Supplementary Figure 4A).

*In vitro* functional imaging of the entire MB shows gradual elevation of Ca^2+^-mediated fluorescence mainly in the lateral neuropils of the MB in response to *Drosophila* ETH (DmETH1) application (Supplementary Figure 4B). We tested the sensitivity of MB γ neurons and DAL neurons to ETH by crossing *MB131B* and *G0431* driver lines (Figure 3B) associated with the strongest memory phenotype with the *UAS-GcaMP5* line. DmETH1 application led to robust Ca^2+^ mobilization in both MB γ lobe and DAL neurons over a 30–40 min period (Figures 3C,C′). Together, these results suggest that ETH targets MB γ lobe and DAL neurons to regulate courtship LTM.

### An ETH-JH Signaling Cascade Regulates Courtship LTM

We previously reported that suppression of ETH signaling through ETHR knockdown specifically in the CA (*JHAMT-GAL4*/*UAS-ETHR RNAi-Sym*) leads to a 70% reduction in JH levels and a consequent reduction of STM performance (Lee et al., 2017). To investigate whether this hormonal cascade also contributes to courtship LTM regulation, we subjected the same genotype to long-term courtship conditioning and found that JH-deficient males show sharply reduced courtship LTM performance without a significant change in courtship activity toward tester virgin females (Figure 4A).

To confirm the necessity of JH in LTM performance, we employed two experimental strategies. We first delayed ETHR knockdown in the CA until 6 days posteclosion by applying the TARGET system (*JHAMT-GAL4*/*UAS-ETHR RNAi-Sym;TubPGAL80^*ts*^*). The objective of this experiment was to allow gene expression-dependent brain maturation to occur prior to alteration of hormonal state. We found that ETHR knockdown in these animals also impaired courtship LTM significantly, whereas both genetic control groups under identical experimental conditions showed normal memory performance (Figure 4B).

We next applied the JH analog methoprene at two doses (1x, 64 pmol and 10x, 640 pmol) to JH-deficient adult males and achieved rescue of memory performance (Figure 4C). The MPI rescue achieved by the higher methoprene dose was not statistically different from that of the GAL4 control. Methoprene treatment had no effect on courtship memory performance of a control group. Thus, the ETH-JH signaling cascade is essential for courtship LTM performance in mature adult males.

Since we have demonstrated that the ETH-JH signaling cascade is required for courtship LTM and that augmented Inka cell activity during the training session improves LTM performance (Figure 1), we also asked whether augmented CA activity through expression of dTrpA1 would enhance courtship LTM *via* increased JH levels. Previous studies showed that Ca^2+^ influx induces biosynthesis and release of JH in the CA (Kikukawa et al., 1987; Yamamoto et al., 1988; Allen et al., 1992; Hsieh et al., 2001; Chiang et al., 2002). We expressed dTrpA1 in the CA using the *JHAMT-GAL4* driver, since this would be expected to depolarize CA cells. However, we and found that, unlike the improvement of memory performance obtained by augmented Inka cell activity, training *JHAMT-GAL4*/*UAS-dTrpA1* males at 31°C did not alter courtship LTM performance (Supplementary Figure 5A). Therefore, improved MPI caused by Inka cell activation (Figure 1) appears not to be mediated by JH elevation, but instead through direct action of ETH on memory circuits. An important caveat associated with the negative result obtained following dTrPA1 expression in CA cells result is that depolarization-dependence of JH release in *Drosophila* has not been demonstrated. To that extent, the negative result reported here remains somewhat equivocal.

### ETH and JH Signaling Converges on MB γ Neurons

Genes encoding two JH receptor paralogs [*Methoprene-tolerant* (*MET*) and *germ cell-expressed* (*GCE*)] are expressed in the adult male brain (Baumann et al., 2010). We performed conditional RNA knockdown of JH receptor genes (*Met* and *gce*) using the *elav-GS-GAL4* (*GSG301*) driver. Since functional redundancy and compensation between these two receptor types have been reported (Baumann et al., 2010; Abdou et al., 2011), we co-expressed RNAi constructs for Met and gce (*UAS-gce RNAi;UAS-Met RNAi*). RU486 treatment of transgenic animals significantly impaired courtship memory performance compared to the vehicle-treated group. We further demonstrated that Met-silencing alone is sufficient for courtship LTM impairment, whereas silencing of the *gce* gene alone results in no significant change in memory performance (Figure 5A).

We previously demonstrated that JH receptor (both MET and GCE) expression in TH (tyrosine hydroxylase)-positive neurons is required for courtship STM performance (Lee et al., 2017). We therefore asked whether these neurons are also involved in regulation of courtship LTM performance by silencing of both *Met* and *gce* genes using the *TH-GAL4* driver. Interestingly, *TH-GAL4*/*UAS-gce RNAi;UAS-Met RNAi* males showed no significant change the memory performance following long-term courtship conditioning (Supplementary Figure 5B). This indicates that, although JH receptors in TH-positive neurons are required for regulation of courtship STM, they are not required for courtship LTM performance.

A recent study of JH receptor expression patterns in the brain of adult *Drosophila* (Baumann et al., 2017) demonstrated strong expression of Met in MB lobes and lateral dorsal neurons (LNd), including DAL neurons. We therefore tested whether Met RNA knockdown in these memory circuit neurons compromises courtship LTM (Figure 5B). Met silencing of the entire MB using the *OK107* driver resulted in strong suppression of LTM performance without gross morphological changes of the MB (Supplementary Figure 3B). Similar to results obtained by silencing *ETHR* genes (Figure 2E), Met silencing in MB γ lobe-containing lines (*201Y* and *1,471*) showed significant memory impairment, whereas silencing Met expression in αβ (*c739*) or α′β′ (*4.59*) line had no effect on courtship LTM performance. Since these GAL4 lines also drive gene expression in additional areas of the brain, specificity of action could be questioned. We therefore performed Met RNA knockdown using the more specific Split-GAL4 MB γ lobe-directed lines *MB009B* and *MB131B* (Figure 3A) and observed significant suppression of courtship LTM. We did not observe memory impairment from Met silencing in DAL neurons. These results indicate that Met expression in the MB γ lobe regulates courtship LTM. In summary, we conclude that converging influences of ETH and JH in this area of the MB is essential for courtship LTM.

## Discussion

We have demonstrated that hormonal state profoundly influences LTM performance in *Drosophila* courtship conditioning. ETH signaling operates both directly and indirectly to regulate memory performance in two distinct ways: (i) directly, by targeting MB γ lobe and DAL neurons; (ii) indirectly *via* allatotropic actions to promote JH production. Joint action of ETH and JH on MB γ lobe neurons is essential for courtship LTM. Augmentation of ETH levels during training enhances memory performance and reduces the minimum training period for LTM. Our findings implicate the joint action of ETH and JH as essential for courtship LTM and that this is independent of the courtship STM pathway described previously (Lee et al., 2017), leading us to propose a model for converging endocrine inputs in the CNS essential for LTM expression following aversive sexual experience (Figure 5C).

### A Direct Endocrine Action for Courtship LTM Modulation: ETH Targets MB and DAL Neurons

Disruption of Inka cell function in adult males impairs memory performance following long-term courtship conditioning. This result, together with loss of memory performance after targeted silencing of ETHR, provides evidence that deficiencies of either ligand or receptor compromise memory performance. These data clearly support the conclusion that ETH signaling is required for memory formation. We reported that the ecdysteroid hormones up-regulate ETH biosynthesis in juvenile (Cho et al., 2014) and adult (Meiselman et al., 2017) *Drosophila*. Ishimoto et al. (2009) found that long-term courtship conditioning leads to increased steroid levels in male flies. We therefore propose that repeated unsuccessful sexual experiences of males induce ETH production through elevation of ecdysteroids. The synthetic origin(s) of ecdysteroids in adult male flies remains unclear.

Augmented Inka cell activity during training enhances features of courtship memory—i.e., learning period and persistence of courtship suppression—without affecting basal courtship activity of naïve males. This suggests that the ETH levels are important not only for driving behavioral change but also for consolidating memory.

Our findings demonstrate direct actions of ETH on two distinct brain circuitries: MB γ lobe and DAL neurons. Several previous studies have revealed that these neural circuits play important roles in post-training memory processes such as consolidation, storage, and retrieval of information (Dubnau et al., 2001; Keleman et al., 2007; Chen et al., 2012; Qin et al., 2012). In particular, expression of Orb2A, a cytoplasmic polyadenylation element-binding (CPEB) protein in the MB γ lobe, is essential for *Drosophila* courtship memory formation (Keleman et al., 2007), by regulating synaptic strength through local protein synthesis in neurons (Kruttner et al., 2012, 2015). The role of synaptic Orb2A is CaMKII-dependent. Therefore, the ETHR-mediated signaling cascade in MB neurons may contribute to memory consolidation through this pathway. However, their courtship conditioning used mated females as tester, which contains anti-aphrodisiac pheromone cues whereas we employed mature virgin females. It is thus not clear whether ETHR-mediated signaling acts *via* Orb2A in MB neurons; this point requires further investigation.

Sakai et al. (2012a) reported that expression of the clock gene *period* (*per*) is essential for retrieval of courtship memory when they used virgin females as tester. Although *per* gene expression in DAL neurons contributes to memory consolidation after olfactory conditioning (Chen et al., 2012), courtship LTM does not require *per* expression in the DAL neurons. In future studies, it would be of great interest to identify interactions between intracellular signaling molecules controlling courtship memory in DAL neurons.

In line with previous reports of ETHR-mediated Ca^2+^ regulation in peptidergic neurons of juvenile flies (Kim et al., 2006; Diao et al., 2016), we find that ETH mobilizes Ca^2+^ in central neurons of mature adult males. We therefore hypothesize that ETHR-mediated intracellular Ca^2+^ signaling in memory circuit neurons provides a key modulatory input for courtship LTM. In a broad range of animal species, Ca^2+^ plays a critical role in memory formation. For example, Ca^2+^ entry through glutamate receptors in mammalian cortical neurons promotes phosphorylation of eEF2, a GTPase that mediates ribosomal translocation along mRNA following peptide bond formation, thereby contributing to long-lasting memory (Belelovsky et al., 2005). The crucial role of cytoplasmic or nuclear Ca^2+^ levels in memory formation has been suggested by several studies in both vertebrates and invertebrates (Meneses, 2007; Perisse et al., 2009; Weislogel et al., 2013). Previous studies also showed that a TRP channel encoded by *painless* (*pain*) (Sakai et al., 2012b) and the small conductance calcium-activated potassium (SK) channel (Abou Tayoun et al., 2012) expressions in the MB neurons are required for *Drosophila* courtship LTM, supporting our hypothesis of the essential role of Ca^2+^ dynamics in formation of this type of memory.

### Convergence of Hormones in Memory Circuits Promotes Courtship LTM

Ecdysis triggering hormone plays another important role in courtship LTM by manipulating JH levels. Suppression of JH production by ETHR silencing in the CA impairs male courtship LTM, while memory impairment can be rescued by the JH analog methoprene, thus demonstrating the essential contribution of the ETH-JH cascade to memory performance. Two observations in this study show that elevation of JH beyond normal levels does not improve memory performance. First, although methoprene rescues courtship LTM of JH-deficient males, high doses do not improve memory performance in either JH-deficient or control groups. Second, unlike the memory enhancement observed following Inka cell activation, reinforced CA function induced by TrpA1 activity during courtship conditioning does not affect LTM. We thus conclude that physiological levels of JH are sufficient for maintenance of memory circuits homeostasis. Our findings also demonstrate that the JH receptor Met is essential for courtship LTM. Importantly, knockdown of Met expression in the MB γ lobe, a target region of ETH, diminishes memory performance. Based on these findings, we propose a hormonal convergence model for courtship LTM (Figure 5C).

In mammals, interactions of neuromodulatory influences within strategic brain areas are essential for emotional memory consolidation. Stress-induced release of glucocorticoids (cortisol in humans) influences memory consolidation *via* both direct and indirect pathways that converge in the amygdala (McGaugh, 2004). In particular, secreted glucocorticoids promote activity of brainstem nuclei (nucleus of the solitary tract, NTS) that connect noradrenergic projections to the basolateral amygdala (BLA), thereby leading to memory formation. Glucocorticoid receptors present in the BLA also facilitate memory consolidation by potentiating noradrenergic signaling through interactions with G protein-mediated cascades.

Although ETH and JH converge on the MB γ lobe to regulate LTM, it remains unclear whether these hormones target the same MB neurons. Furthermore, even if they do target the same neurons, how signal transduction and possible new gene expression resulting from their actions contributes to LTM remain to be elucidated. As discussed, G protein-mediated Ca^2+^ signaling is critical for memory formation through diverse downstream mechanisms. Previous reports showing that JH promotes protein phosphorylation in moths and mosquitoes (Arif et al., 2002; Liu et al., 2015), provides some insights into possible joint actions of ETH-mediated GPCR signaling and JH-mediated Met function for promotion of diverse cellular responses. For example, ETHR-mediated Ca^2+^ mobilization may combine with JH receptor-mediated protein phosphorylation to enhance presynaptic protein synthesis. It will be important to investigate the detailed mechanisms underlying ETH- and JH-mediated signaling at the molecular level.

Previously, there has been a presumption that LTM is sequentially formed following the STM process (McGaugh, 1966). However, studies have reported that the circuit-based mechanisms underlying STM and LTM in diverse animal models can be independent and parallel (Botzer et al., 1998; Izquierdo et al., 1998, 1999; Blum et al., 2009; Sanderson et al., 2009; Trannoy et al., 2011; Yamagata et al., 2015). Our previous study showed that expression of JH receptors in dopaminergic (DA) neurons is essential for courtship STM (Lee et al., 2017). The function of JH in these neurons likely contributes to maintenance of information, even during the short-term interval following aversive experience. Nevertheless, we found in the current study that silencing of JH receptors in DA neurons does not affect courtship LTM performance. Instead, Met expression in the MB γ lobe neurons is critical for LTM. Thus, our findings argue for parallel pathways in regulation of courtship STM and LTM through independent neuronal targets of endocrine signals ETH and JH.

In summary, we have identified novel hormonal pathways that regulate 24-h lasting LTM following their induction by extended courtship conditioning in *Drosophila*. ETH modulates memory directly through Ca^2+^-mediated mechanisms in the MB lobe and DAL neurons, and indirectly through regulation of JH production. Furthermore, ETH and JH cascades converge in a functionally-distinct area of the MB that is critical for courtship LTM. Thus, we provide a testable model for the essential involvement of endocrine state in the formation of context-dependent social memories.

## Data Availability Statement

The raw data supporting the conclusions of this article will be made available by the authors, without undue reservation.

## Author Contributions

SL performed behavioral assays, immunohistochemistry, and functional Ca^2+^ imaging. Both authors designed the experiments and wrote the manuscript.

## Conflict of Interest

The authors declare that the research was conducted in the absence of any commercial or financial relationships that could be construed as a potential conflict of interest.

## Footnotes

1 https://splitgal4.janelia.org/cgi-bin/splitgal4.cgi
