# Supplementary material for: Regulation of Drosophila Long-Term Courtship Memory by Ecdysis Triggering Hormone
Source: Front Neurosci. 2021 Apr 22;15:670322. doi: 10.3389/fnins.2021.670322 (PMC8100193; doi:10.3389/fnins.2021.670322)
Supplement: Supplementary file 1 [file Data_Sheet_1.pdf]

Supplementary Material

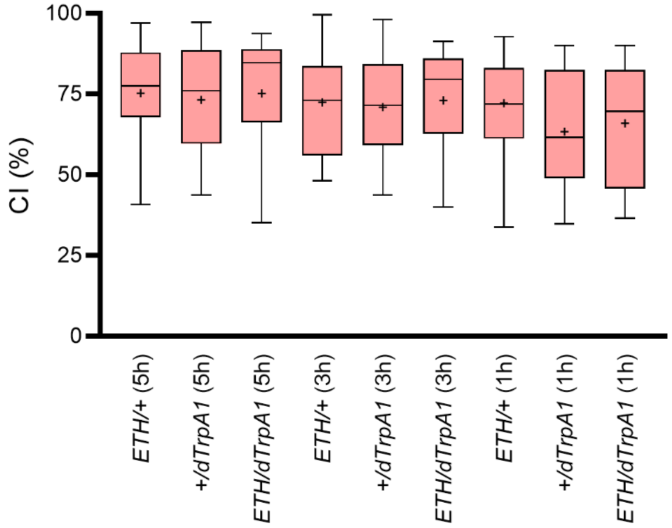

Figure S1. Activation of Inka cells during training sessions (5-, 3-, and 1-hour heat activation) does not alter basal courtship activity of sham-trained males toward immobilized virgin females during 10-min testing sessions. No significant change in courtship index (CI) was observed compared to genetic control groups under identical experimental conditions (n = 33-40). Mann-Whitney *U* Test.

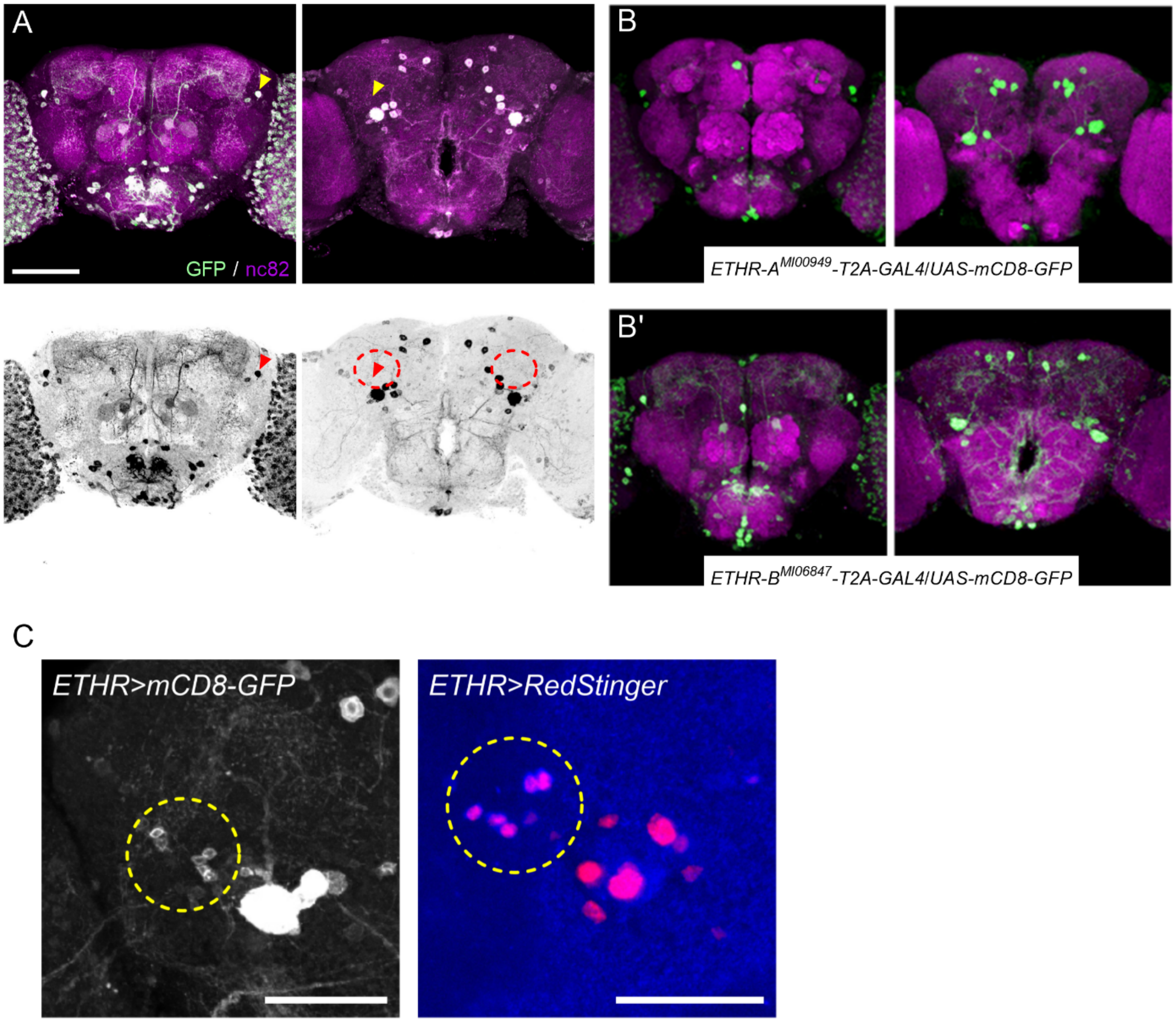

Figure S2. ETHR is expressed in broad areas of adult male brain. **(A)** Day-4 male brain of an *ETHR-GAL4* line (*MH1506*) expressing GFP (*UAS-mCD8-GFP*). This line drives to cells expressing both ETHR-A and ETHR-B subtypes (Diao et al., 2016). Anterior (left) and posterior (right). Arrowheads indicate putative DAL neuron (left) and Kenyon cell areas (right). A putative calyx area is indicated as dashed circles. Scale bar represents 100 µm. **(B and B′)** Expression of ETHR subtypes in day-4 male brain. ETHR-A **(B)** and ETHR-B **(B′)** expression in anterior (left) and posterior (right) brain sides. **(C)** An *ETHR-GAL4* driver line (*MH1506*) labels a small population of Kenyon cell-like neurons. Left: posterior view of the *ETHR-GAL4*/*UAS-mCD8-GFP* male; right, posterior view of an *ETHR-GAL4*/*UAS-RedStinger* male. Yellow circles represent area of interest. Scale bars = 20 µm.

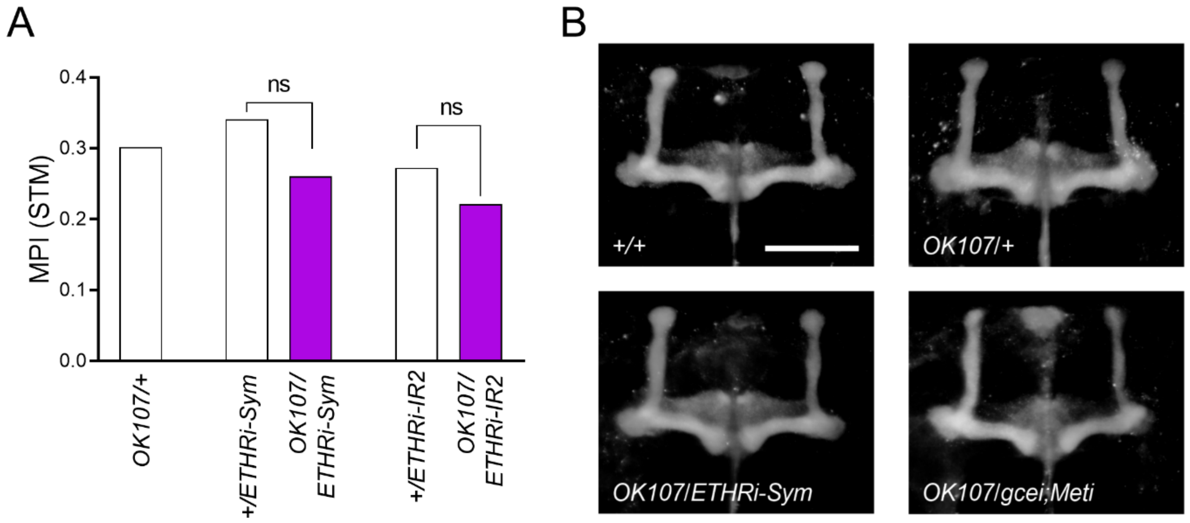

Figure S3. (A) The role of ETHR expression in the MB for STM was tested by silencing ETHR genes in the entire MB (*OK107*/*UAS-ETHR RNAi-Sym* and *OK107*/*UAS-ETHR RNAi-IR2*). A male was paired with a mated female for 1 hour. Following one 10-min interval, memory performance was tested by pairing the conditioned male with an immobilized virgin female for 10 minutes (n = 50-54). Random Permutation Test, ns, no significance. **(B)** RNA knockdown of ETHR or JH receptors in the MB (*OK107*, entire MB) does not induce gross morphological changes. Brains of day 4-5 posteclosion males were stained against Fas-II to visualize the MB. Scale bar represents 50 µm.

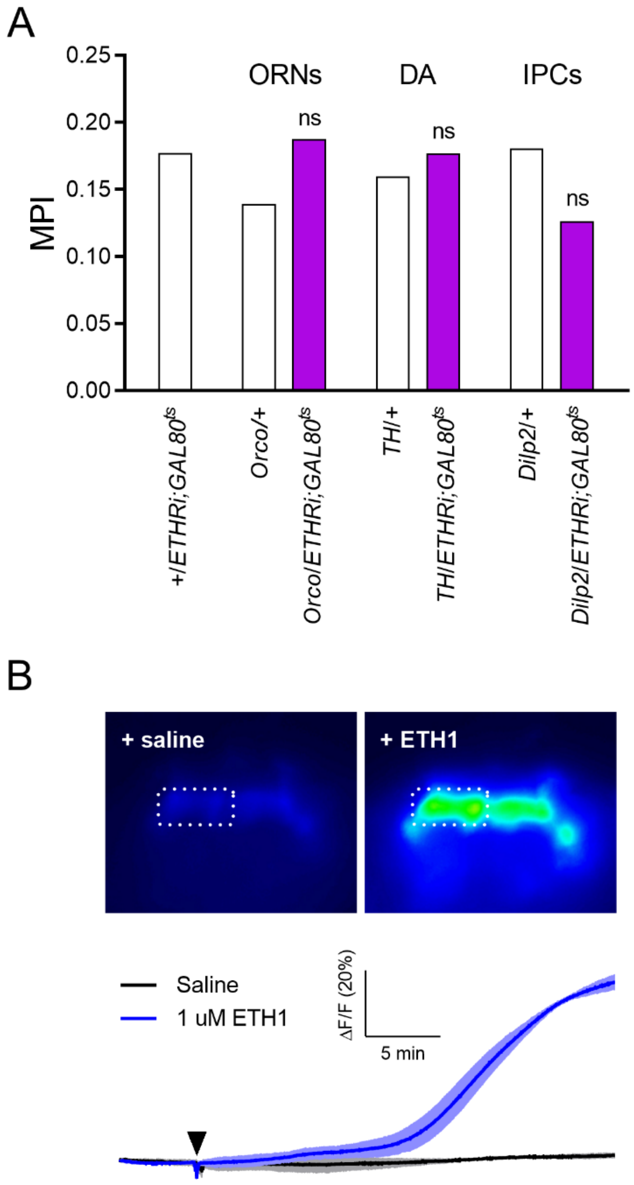

Figure S4. (A) Conditional ETHR silencing in ORNs, TH-positive DA neurons, and IPCs in the brain does not impair courtship LTM. Training was conducted by housing individual males at 31°C during adulthood except during training and testing sessions (n = 54-60). Random Permutation Test, ns, no significance. **(B)** ETH mobilizes Ca^{2+} in MB lateral neuropils: above, Ca^{2+}-dependent fluorescence in MB lateral neuropil area of a *OK107/UAS-GCaMP5* male brain in response to 1 μM DmETH1 in the absence (left) and presence (right) of ETH; bottom, averaged traces of percent $\Delta F/F_0$ following saline control (black) and 1 μM DmETH1 (blue) treatments. Arrowhead represents saline or ETH application. Darker lines represent mean, while the light envelopes represent SEM (n = 3 for each).

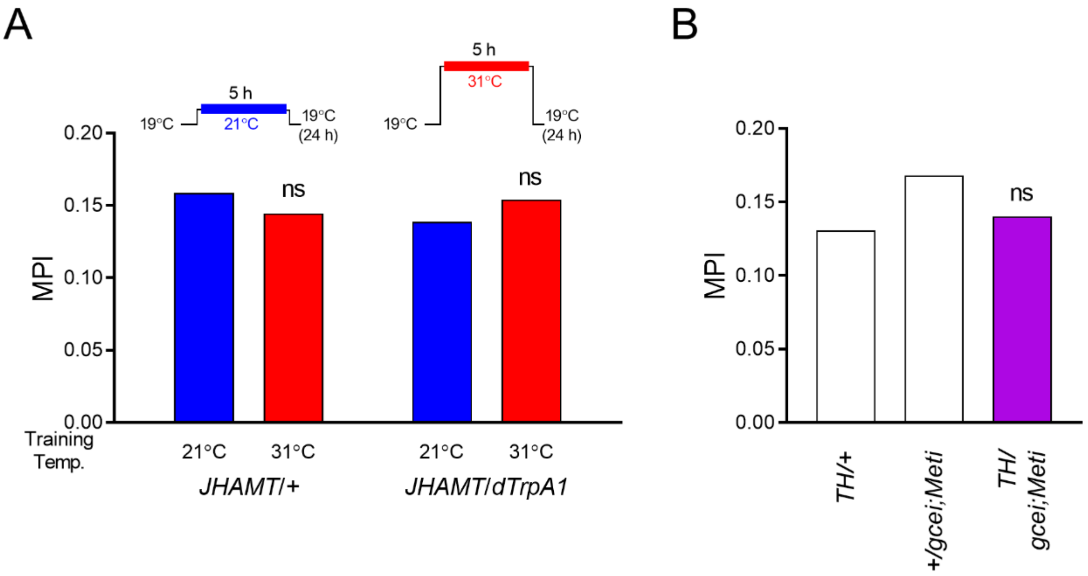

Figure S5. (A) CA stimulation during courtship conditioning does not affect LTM performance. Individual males were placed at 21℃ or 31℃ for 5 hours with a mated female and tested for courtship suppression after a 24-hour interval at 21℃ compared to CI of sham-trained males (n = 54-64). **(B)** RNA knockdown of both JH receptors (MET and GCE) in TH-positive neurons does not affect 24-hour lasting memory following 5-hour training with a mated female (n = 52-60). Random Permutation Test, ns, no significance.
